# Advance care planning in primary malignant brain tumors: Knowledge, experiences, and preferences of patients and caregivers

**DOI:** 10.1093/nop/npaf008

**Published:** 2025-01-21

**Authors:** Ai Chikada, Yoshiki Arakawa, Sayaka Takenouchi, Yoshitaka Narita

**Affiliations:** Human Health Sciences, Graduate School of Medicine, Kyoto University, Sakyo-ku, Kyoto, Japan; Department of Neurosurgery, Kyoto University Graduate School of Medicine, Sakyo-ku, Kyoto, Japan; Human Health Sciences, Graduate School of Medicine, Kyoto University, Sakyo-ku, Kyoto, Japan; Department of Neurosurgery and Neuro-Oncology, National Cancer Center Hospital, Chuo-ku, Tokyo, Japan

**Keywords:** advance care planning, caregiver, decision-making, patient engagement, primary malignant brain tumors

## Abstract

**Background:**

Advance care planning (ACP) can help patients with primary malignant brain tumors to align treatments with their preferences. However, insights into patients’ and caregivers’ engagement with ACP remain scarce. This study elaborates on their knowledge, experiences, and preferences concerning ACP.

**Methods:**

This was a secondary analysis of data from the “National Survey on the Needs and Support of Brain Tumor Patients and Caregivers” in Japan. Responses from 128 patients and 106 caregivers were included. Descriptive statistics, logistic regression, and qualitative analyses of free-text responses were performed.

**Results:**

Patients were more willing than caregivers to participate in decisions regarding their treatment (96.8% vs. 82.5%, *P* < .001). Knowledge about ACP was low in both groups (12.3% of patients and 10.7% of caregivers), but willingness to participate in ACP was high (68.9% of patients and 65.9% of caregivers). Preference to initiate ACP at diagnosis was low in both groups, with caregivers showing a higher preference than patients (29.0% vs. 11.1%). A greater percentage of patients preferred to start ACP at recurrence than caregivers (47.0% vs. 18.3%, *P* < .0001). Frequent family discussions were significantly associated with actual experiences of ACP (OR = 3.7, 95% CI = 1.6–9.3, *P* = .0019).

**Conclusions:**

The mismatch between respondents’ willingness to participate in ACP and their knowledge and experience reveals a need to increase ACP awareness. Differences in ACP preferences between patients and their caregivers may indicate the need for improved communication strategies by healthcare professionals. Further research is needed to understand these differences.

**Key Points**

1. Healthcare professionals aid advance care planning among patients and caregivers.
2. Family discussions align treatment with patient values in advance care planning.

**Importance of the Study**

This study highlights key differences in the preferences and attitudes of patients with primary malignant brain tumors and their caregivers regarding advance care planning (ACP) in Japan. While patients showed a preference for delaying ACP discussions until disease progression, caregivers preferred initiating these conversations earlier. Despite low ACP knowledge between the 2 groups, the willingness to engage in ACP was high, especially when prompted by healthcare professionals (HCPs). Frequent family discussions were also found to be crucial in facilitating ACP. The findings underscore the need for increased awareness and proactive involvement from HCPs in promoting ACP, as early engagement can improve the alignment between treatment and patient values, ultimately enhancing patient-centered care. Future research should aim to understand the reasons behind these differences and develop targeted strategies to improve engagement in ACP among patients and caregivers, supporting timely and meaningful discussions.

Advance care planning (ACP) is defined as discussions between individuals and their families, supported by healthcare professionals (HCPs) with whom they have a trusting relationship to prepare for their way of life, medical treatment, and care preferences.^1^ It is a critical component of comprehensive, patient-centered healthcare,^2^ particularly for individuals with life-limiting conditions such as primary malignant brain tumors (PMBTs).^3^ The diagnosis of a PMBT often marks the onset of a tumultuous journey for patients and their families.^4^ Confronted with the labyrinth of treatment choices, prognostic uncertainties, and the potential for rapid progression associated with PMBT morbidity, ACP can offer a sense of control and preparedness.^5,6^ It allows patients to articulate their preferences for end-of-life care, should they become unable to communicate their decisions.^7,8^ More importantly, it fosters open communication between patients, their families, and HCPs, aligning care with the patient’s values and wishes.^7^

Despite the recognized importance of ACP in the context of PMBTs, conversations around ACP tend to occur late in the disease trajectory.^9^ This delay is often attributable to patients’ and families’ ongoing expectations for antitumor therapy,^10^ as well as HCPs’ knowledge and time constraints.^11^ Additionally, the ACP process can induce stress in both patients^12^ and HCPs,^13^ and family members may unintentionally dominate ACP discussions.^14^ These factors collectively contribute to the suboptimal effectiveness of ACP implementation. In Japan, the Ministry of Health, Labour and Welfare (MHLW) began promoting the term “advance care planning,” locally known as “人生会議” (*jinseikaigi*, which means “meeting about life”), through various media outlets in 2018. However, effective implementation of ACP in Japan is complicated by Japanese cultural and systemic factors, such as taboos surrounding death and the universal healthcare system that ensures access to medical treatment for as long as desired.

Although previous research has highlighted the significance of ACP participation and identified various barriers experienced by patients with PMBTs, a significant knowledge gap remains regarding patients’, families’, and caregivers’ actual understanding, experiences, and preferences regarding ACP.

The present study aims to fill this gap, analyzing and describing patients’ and caregivers’ knowledge, experiences, and preferences regarding ACP and thereby providing a more comprehensive understanding of the nuances of ACP engagement among people with PMBTs.

## Materials and Methods

### Study Design

This study was a secondary analysis of data originally collected via the “National Survey on the Needs and Support of Brain Tumor Patients and Caregivers,” a web-based questionnaire conducted between April and December 2023 by the Committee on Supportive Care for Brain Tumor Patients (SCBTP) under the Japan Society for Neuro-Oncology (JSNO). The present authors participated in this original study, which received ethical approval from the National Cancer Center Research Ethics Review Board (2022-430). While the initial survey aimed to broadly understand the experiences and needs of patients with PMBTs and their caregivers, the present research narrowed its focus to exploring aspects of ACP. By selectively extracting and analyzing subsets of data pertinent to ACP, this secondary analysis sought to uncover deeper insights into this population’s knowledge, experiences, and preferences concerning ACP.

The participants provided consent electronically via the web-based survey form. Before proceeding with the survey, they were asked to confirm their agreement with the study’s objectives and data usage. They could either agree and proceed or decline, which would terminate the survey. They were also informed that they could stop the survey at any time before submission, and their responses would be cleared. Additionally, they were informed that once their responses were submitted, withdrawal of their data would no longer be possible, as the data could not be traced back to them.

### Recruitment and Participants

Prior to conducting the original survey, we distributed survey request forms to physicians affiliated with university hospitals, cancer centers, designated cancer hospitals, and other institutions in Japan that manage a significant number of brain tumor cases. Recruitment was conducted through neurosurgeons, who were asked to distribute the survey request forms to inpatients, outpatients, and their caregivers. In Japan, neurosurgeons often fulfill the role of neuro-oncologists and are typically involved in the care of patients with brain tumor from the time of diagnosis, and they manage both surgical and nonsurgical aspects of treatment, including supportive care and end-of-life care. The survey was promoted via websites and social media platforms associated with the First Annual Meeting of SCBTP, the National Cancer Center’s Rare Cancer Center, and the Japan Brain Tumor Alliance.

The resulting data set included individuals diagnosed with PMBTs who were aged 18 years or older, as well as their caregivers—including family members, partners, acquaintances, or others involved in the patient’s care. Participants from both groups were required to have a comprehensive understanding of the survey’s contents, as detailed in the survey request form, and they voluntarily consented to participate. No exclusion criteria were applied. Data related to the participants’ age were collected using broader categories (eg, under 10, 10s, 20s, etc.). Consequently, the participants aged 20 and above, including both patients diagnosed with PMBTs and their caregivers, were extracted in this second analysis.

### Questionnaire Development and Implementation

The questionnaire for the original study was developed through a meticulous, iterative process by the SCBTP’s ACP Working Group. This committee included a diverse group of HCPs —neurosurgeons, rehabilitation doctors, radiologists, psychologists, nurses, physical therapists, and occupational therapists, among others—as well as patients and their family members, embodying a multidisciplinary approach. The questionnaire, which was divided into 2 Google Forms for patients and their caregivers, respectively, encompassed a wide range of topics related to treating and living with PMBTs. It included questions about treatment progress, demographic information, and health and psychosocial status, with specific sections focusing on medical conditions and respondents’ experiences, perspectives, and needs for treatment, rehabilitation, medical explanations, and ACP. There was no link between the patients’ answers and their caregivers’ answers.

The Distress and Impact Thermometer (DIT), a brief screening tool for adjustment disorders and depression, was also included in this study.^15^ The DIT consists of 2 scales with scores ranging from 0 to 10: 1 scale measures distress, while the other measures the impact of distress on daily life. A cutoff score of 4 or higher for distress and 3 or higher for impact was established to detect adjustment disorders or depression in a previous study involving 295 cancer patients, which demonstrated a sensitivity of 0.82 and a specificity of 0.82.^15^ In this study, we used the DIT to capture group-level psychological trends rather than for individual diagnosis or intervention.

ACP experience was assessed using a question modeled after the Japanese MHLW’s national survey on end-of-life care preferences, which is conducted every 5 years among the general population. The participants were asked whether they had discussed treatments and care at the end of life, with response options of “In-depth discussion,” “Some discussion,” or “No discussion.” For the analysis, we converted the responses into binary data by grouping “In-depth” and “Some discussion” as “Yes” and “No discussion” as “No.”

For this study, we extracted the responses related to medical conditions and ACP, along with the participants’ demographic information.

### Statistical Analysis

Descriptive statistics were used to summarize the data. Associations between categorical variables were examined by conducting a chi-squared test, with Fisher’s exact test conducted as required for smaller sample sizes. When comparing ACP-related factors between the patients and caregivers (as presented in Table 2), these tests were conducted without controlling for covariates, to descriptively highlight the differences in perspectives and experiences rather than to predict outcomes. For logistic regression analysis, the variables were converted into binary data to assess the impact of frequent family discussions on actual patients’ ACP experience. This analysis was adjusted for factors such as age, treatment facility for brain tumors, and surgical history of brain tumors to control for potential confounding effects.

**Table 2.** Comparison of ACP Factors Related to the Patients and Caregivers

Variable, *n* (%)	Patient	Caregiver	*P*-Value
Understanding of the patients’ medical condition			
Sufficient	113 (89.7)	100 (96.2)	.074^†^
Insufficient	13 (10.3)	4 (3.9)	
Total	126	104	
Satisfaction with information provided by healthcare professionals			
Yes	64 (50.8)	43 (42.2)	.193
No	62 (49.2)	59 (57.8)	
Total	126	102	
The patients’ preference for participation in decision-making regarding medical treatment and care			
Yes	120 (96.8)	85 (82.5)	<.001*^†^
No	4 (3.2)	18 (17.5)	
Total	124	103	
Frequency of discussion regarding treatment and care among the patients and caregivers			
Voluntary	85 (68.0)	62 (60.8)	.258
Compulsory	40 (32)	40 (39.2)	
Total	125	102	
Experience in discussing life-prolonging therapeutic treatment before being diagnosed with PMBTs			
Yes	31 (24.8)	31 (30.7)	.324
No	94 (75.2)	70 (69.3)	
Total	125	101	
Knowledge of ACP			
Yes	15 (12.3)	11 (10.7)	.705
No	107 (87.7)	92 (89.3)	
Total	122	103	
Experience with ACP			
Yes	53 (43.1)	30 (29.7)	.038*
No	70 (56.9)	71 (70.3)	
Total	123	101	
Willingness to engage in ACP			
Yes	71 (68.9)	60 (65.9)	.656
No	32 (31.1)	31 (34.1)	
Total	103	91	
Sharing the patients’ values with the surrogate decision-maker			
Yes	55 (44.4)	30 (33.0)	.09
No	69 (55.7)	61 (67.0)	
Total	114	91	

Abbreviations: ACP, advance care planning; PMBTs, primary malignant brain tumors. Footnote: First, *n* = 128 and *n* = 106 refer to the total number of patients and caregivers, respectively, who responded to the survey. Some items may have missing responses, and the total number of participants may vary between variables. Therefore, the actual number of patients and caregivers for each variable is indicated. Second, statistical tests performed included a chi-square test for variables with sufficient expected frequencies and

^†^Fisher’s exact test for variables with small expected frequencies. Significant *P*-values (*P *< .05) are marked with an asterisk (*).

All statistical analyses were performed using JMP® Pro 17 (SAS Institute Inc.), with a significance level set at less than 5%.

### Qualitative Analysis

Free-text responses were analyzed by conducting qualitative inductive analysis to categorize recurring themes and uncover dominant attitudes toward ACP. This analysis adhered to the methods described by Braun and Clarke (2006) for thematic analysis, ensuring a systematic coding of responses into relevant categories.^16^ To provide context and highlight the prominence of certain themes, frequency counts were incorporated following Krippendorff’s content analysis approach.^17^

The participants first selected 1 of 4 single-choice options in response to the question, “What do you think about ACP?” Then, they provided free-text responses. The qualitative analysis focused on identifying themes from these free-text responses, which were then categorized into subcategories representing common themes. The “n” in the subcategory column of Appendix Tables 1 and 2 indicates the number of free-text responses classified into each subcategory. The subcategories in this study were designed to reflect the individual perspectives of the participants without overgeneralizing their views. This approach ensured a detailed understanding of both patients’ and caregivers’ preferences regarding ACP, capturing the nuances of the discussion.

To ensure rigor during the coding/categorization process, the analysis was supervised by a palliative care nursing researcher with extensive qualitative expertise.

## Results

**Figure 1.**
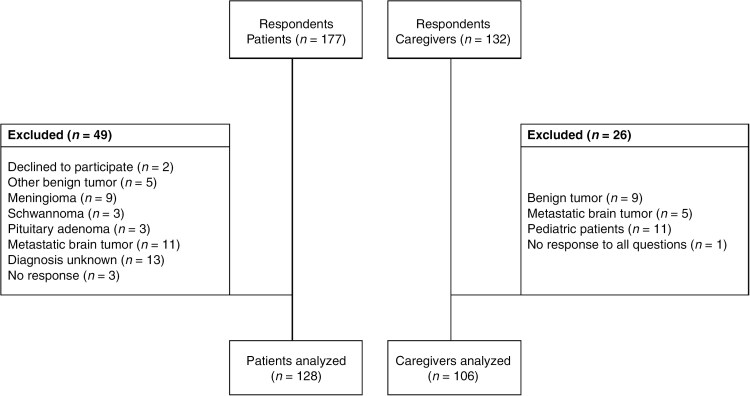
Flowchart for the selection of study participants. (Left-hand side) Patient exclusion criteria. (Right-hand side) Caregiver exclusion criteria.

In total, 177 patients and 132 caregivers responded to the original survey. After applying the exclusion criteria, 128 patients and 106 caregivers were included in the present analysis, as shown in Figure 1. Table 1 presents the demographic and clinical characteristics of the patients and their caregivers.

**Table 1.** Demographic and Clinical Characteristics of the Study Participants

Variables	Patients (*n* = 128)	Caregivers (*n* = 106)	*P*-Value
	*n* (%)	*n* (%)	
Age group			.009
20s	7 (5.5)	1 (1.0)	
30s	16 (12.6)	10 (9.6)	
40s	41 (32.3)	18 (17.3)	
50s	35 (27.6)	33 (31.7)	
60s	18 (14.2)	28 (26.9)	
70s	9 (7.1)	14 (13.5)	
80s	1 (0.8)	0 (0.0)	
Not stated	1	2	
Sex			<.001
Male	76 (60.3)	36 (34.0)	
Female	50 (39.7)	70 (66.0)	
Not stated	2	0	
Age group of the care recipients			
20s	N/A^a^	14 (13.2)	
30s	N/A	7 (6.6)	
40s	N/A	19 (17.9)	
50s	N/A	17 (16.0)	
60s	N/A	27 (25.5)	
70s	N/A	18 (17.0)	
80s	N/A	4 (3.8)	
Sex of the care recipients			
Male	N/A	65 (61.3)	
Female	N/A	41 (38.7)	
Relationship with the patient			
Spouse/partner	N/A	69 (65.1)	
Parent	N/A	19 (17.9)	
Child	N/A	12 (11.3)	
Sibling	N/A	5 (4.7)	
Other (cousin or other peer)	N/A	1 (0.9)	
Primary caregiver and surrogate decision-maker regarding the patients’ medical treatment and care			
Yes	N/A	89 (93.7)	
No	N/A	6 (6.3)	
Not stated	N/A	11	
Duration since brain tumor diagnosis			.487
Less than 6 months	13 (10.2)	10 (9.4)	
6 months to less than 1 year	10 (7.8)	16 (15.1)	
1 year to less than 2 years	19 (14.8)	20 (18.9)	
2 years to less than 5 years	40 (31.3)	29 (27.4)	
5 years to less than 10 years	25 (19.5)	17 (16.0)	
10 years or more	21 (16.4)	14 (13.2)	
Treatment facility for brain tumors			.118
University hospital	64 (50.4)	47 (44.3)	
Cancer center	56 (44.1)	45 (42.5)	
Other hospitals	7 (5.5)	14 (13.2)	
Patient’s surgical history of brain tumors			.726
Yes	122 (95.3)	99 (93.4)	
No	6 (4.7)	7 (6.6)	
Types of primary malignant brain tumors			.117
Glioblastoma	43 (33.6)	48 (45.3)	
Glioma grade 3	34 (26.6)	27 (25.5)	
Glioma grade 2	36 (28.1)	11 (10.4)	
Primary central nervous system lymphoma	5 (3.9)	5 (4.7)	
Other malignant brain tumors	10 (7.8)	15 (14.2)	
Patient’s KPS^b^			<.001
≥70	113 (88.3)	61 (57.5)	
<70	15 (11.7)	45 (42.5)	
Distress and Impact Thermometer			.009
Positive	29 (23.0)	41 (39.8)	
Negative	97 (77.0)	62 (60.2)	

^a^“N/A” for missing data.

^b^Patient’s KPS, as reported by caregivers, is based on the caregiver’s observations of the patient’s activities of daily living.

Table 2 presents a comparative analysis of the factors related to decision-making between the patients and caregivers. Significant differences were noted in several areas: the patients were more likely than the caregivers to prefer participating in decision-making regarding their medical treatment and care (96.8% vs. 82.5%, *P* < .001). Additionally, the patients were more likely than the caregivers to have experienced ACP (43.1% vs. 29.7%, *P* = .038). Knowledge of ACP was low in both groups, with only 12.3% of the patients and 10.7% of the caregivers reporting awareness (*P* = .705). Despite these low knowledge levels, willingness to engage in ACP was relatively high among both patients (68.9%) and caregivers (65.9%) (*P* = .656). No significant differences were found between the patients and caregivers regarding their understanding of the patients’ medical conditions (*P* = .074), their satisfaction with the information provided by HCPs (*P* = .193), the frequency of discussions regarding treatment and care (*P* = .258), their experience discussing life-prolonging therapeutic treatment before diagnosis (*P* = .324), or their willingness to communicate the patients’ values to a surrogate decision-maker (*P* = .090).

Timing preferences for when to initiate ACP are presented in Table 3. The chi-squared test showed a statistically significant difference in the patients’ and caregivers’ timing preferences (χ² = 24.9, *df* = 4, *P* < .0001). The residual analysis indicated that the significant difference was primarily due to the caregivers’ higher preference to initiate ACP at the time of diagnosis (standardized residual = 2.31, *P* = .02) and the patients’ higher preference to initiate ACP upon recurrence of the brain tumor (standardized residual = 2.35, *P* = .02). Only 11.1% of the patients favored initiating ACP at the time of diagnosis, compared to 29.0% of the caregivers. In contrast, the majority of the patients (47.0%) preferred to start ACP when the brain tumor recurred, compared to only 18.3% of the caregivers. Additionally, 20.5% of the patients and 19.6% of the caregivers preferred initiating ACP when palliative care became the primary focus; 12.8% of the patients and 20.4% of the caregivers preferred to defer to the HCP’s suggested timing; and 8.6% of the patients and 12.9% of the caregivers preferred some other timing.

**Table 3.** Patients’ and Caregivers’ Preferred Timing for Initiating ACP

Timing Preference	Patients (*n* = 117)		Caregivers (*n* = 93)	
	*n*	%	*n*	%
When diagnosed with the illness	13	11.1	27	29.0
When the brain tumor recurs	55	47.0	17	18.3
When palliative care is primary	24	20.5	18	19.6
When deemed necessary by HCPs^a^	15	12.8	19	20.4
Other	10	8.6	12	12.9

Chi-squared tests showed a statistically significant difference in patients’ and caregivers’ timing preferences for initiating ACP (χ² = 24.9, *df* = 4, *P* = <.0001).

Abbreviation: ACP, advance care planning.

^a^HCPs, health care professionals.

Table 4 presents the demographic differences in decision-making among the patients with PMBTs, analyzed by conducting a chi-square test or Fisher’s exact test, where appropriate. Significant differences were noted in several variables: younger patients and those with higher KPS measures were significantly more likely to share advance directives (ADs) with their families (*P* = .0168 and *P* = .0111, respectively) than older or lower-scoring patients. The patients with a history of surgery for brain tumors were also significantly more likely to share ADs with their families (*P* = .0406). The patients who reported previous discussions about life-prolonging therapeutic treatments before diagnosis were significantly more likely to have experience with ACP and to share ADs with their families (*P* < .0001 for both). Furthermore, voluntary discussions between the patients and family members regarding treatment and care were significantly associated with higher ACP experience and more AD sharing (*P* = .0017 and *P* = .0002, respectively).

**Table 4.** Comparison of Patient Characteristics and Clinical Features and Knowledge, Experience, and Willingness to Engage in ACP and Sharing of ADs

Variables	Knowledge of ACP			Experience With ACP			Willingness to Engage in ACP			AD Shared With Family		
	Yes	No	*P*-Value	Yes	No	*P*-Value	Yes	No	*P*-Value	Yes	No	*P-*Value
Age group												
60s and younger	14 (11.5)	98 (80.3)	1.0000^†^	47 (38.6)	65 (53.3)	.3279^†^	67 (62.6)	31 (29.0)	1.0000^†^	44 (35.4)	70 (56.5)	.0168*^†^
70s and older	1 (0.8)	9 (7.4)		6 (4.9)	4 (3.3)		6 (5.6)	3 (2.8)		8 (6.5)	2 (1.6)	
Treatment facility for brain tumors												
University hospital or cancer center	13 (10.7)	102 (83.6)	.2060^†^	49 (40.2)	66 (54.1)	.4665^†^	70 (65.4)	32 (29.9)	.6519^†^	49 (39.5)	68 (54.8)	1.00
Other hospitals	2 (1.6)	5 (4.1)		4 (3.3)	3 (2.5)		3 (2.8)	2 (1.9)		3 (2.4)	4 (3.2)	
Brain tumor type												
Glioma^a^	13 (10.6)	97 (78.9)	.6594^†^	47 (38.2)	62 (50.4)	1.0000^†^	67 (62.0)	29 (26.9)	.1975^†^	47 (37.6)	64 (51.2)	.7766^†^
Non-glioma (malignant)^a^	2 (1.6)	11 (8.9)		6 (4.9)	8 (6.5)		6 (5.6)	6 (5.6)		5 (4.0)	9 (7.2)	
History of surgery for brain tumors												
Yes	14 (11.4)	103 (83.7)	.5497^†^	52 (42.3)	65 (52.9)	.2435^†^	69 (63.9)	34 (31.5)	1.0000^†^	52 (41.6)	0 (0)	.0406*^†^
No	1 (0.8)	5 (4.1)		1 (0.8)	5 (4.1)		4 (3.7)	1 (0.9)		67 (56.3)	6 (4.8)	
KPS												
≥70	14 (11.4)	94 (76.4)	.6916^†^	43 (35.0)	65 (52.9)	.0569^†^	65 (60.2)	31 (28.7)	1.0000^†^	41 (32.8)	69 (55.2)	.0111*^†^
<70	1 (0.8)	14 (11.4)		10 (8.1)	5 (4.1)		8 (7.4)	4 (3.7)		11 (8.8)	4 (3.2)	
Distress and Impact Thermometer												
Positive	1 (0.8)	27 (22.0)	.1869^†^	10 (8.1)	18 (14.6)	.3667	14 (13.0)	8 (7.4)	.7990^†^	14 (11.2)	15 (12.0)	.4072
Negative	14 (11.4)	81 (65.9)		43 (35.0)	52 (42.3)		59 (54.6)	27 (25.0)		38 (30.4)	58 (46.4)	
Patients’ preference for participation in decision-making regarding medical treatment and care												
Yes	15 (12.4)	103 (85.1)	1.0000^†^	49 (40.5)	68 (56.2)	1.0000^†^	71 (67.0)	33 (31.1)	.5407	49 (39.8)	70 (56.9)	.6454^†^
No	0 (0.0)	3 (2.5)		2 (1.7)	2 (1.7)		1 (0.9)	1 (0.9)		1 (0.8)	3 (2.4)	
Preference regarding prognosis disclosure												
Yes	13 (10.6)	86 (69.9)	.7332^†^	44 (35.8)	55 (44.7)	.6479^†^	63 (58.3)	25 (23.2)	.0693	42 (33.6)	59 (47.2)	.9941
No	2 (1.6)	22 (17.9)		9 (7.3)	15 (12.2)		10 (9.3)	10 (9.3)		10 (8.0)	14 (11.2)	
Understanding of the patients’ medical condition												
Sufficient	15 (12.2)	95 (77.2)	.3645^†^	47 (38.2)	63 (51.2)	1.0000^†^	66 (61.1)	31 (28.7)	.7450^†^	47 (37.6)	65 (52.0)	1.0000^†^
Insufficient	0 (0.0)	13 (10.6)		6 (4.9)	7 (5.7)		7 (6.5)	4 (3.7)		5 (4.0)	8 (6.4)	
Satisfaction with information provided by healthcare professionals												
Yes	6 (4.9)	55 (44.7)	.5831^†^	26 (21.1)	36 (29.3)	.7944	38 (35.2)	15 (13.9)	.3702	27 (21.6)	36 (28.8)	.7737
No	9 (7.3)	53 (43.1)		27 (22.0)	34 (27.6)		35 (32.4)	20 (18.5)		25 (20.0)	37 (29.6)	
Experience discussing life-prolonging therapeutic treatment before being diagnosed with PMBTs												
Yes	5 (4.1)	25 (20.3)	.5205^†^	22 (17.9)	7 (5.7)	<.0001*^†^	17 (15.7)	9 (8.3)	.8127*	26 (20.8)	5 (4.0)	<.0001*^†^
No	10 (8.1)	83 (67.5)		31 (25.2)	63 (51.2)		56 (51.9)	26 (24.1)		26 (20.8)	68 (54.4)	
Frequency of the patients’ and caregivers’ discussions regarding treatment and care												
Voluntary	8 (6.5)	76 (61.8)	.2369^†^	44 (35.8)	39 (31.7)	.0017*^†^	53 (49.1)	20 (18.5)	.1118	45 (36.0)	40 (32.0)	.0002*^†^
Compulsory	7 (5.7)	32 (26.2)		9 (7.3)	31 (25.2)		20 (18.5)	15 (13.9)		7 (5.6)	33 (26.4)	

Abbreviations: ACP, advance care planning; ADs, advance directives; HGG, high-grade glioma; PMBTs, primary malignant brain tumors.

^a^Glioma includes glioblastoma, grade 3 glioma, and grade 2 glioma. Non-glioma (malignant) includes primary central nervous system lymphoma and other malignant brain tumors.

^†^Statistical tests performed included a chi-square test for variables with sufficient expected frequencies and Fisher’s exact test for variables with small expected frequencies. Significant *P*-values (*P* < .05) are marked with an asterisk (*).

Table 5 presents the results of the logistic regression analyses, which assessed whether the frequency of family discussions about the patients’ treatment and care impacted their actual experience with ACP. In the crude model, “Discussion with family” was significantly associated with actual ACP experience (β = −0.6, SE = 0.2, OR = 3.9, 95% CI = 1.7–9.6, *P* = .001). In the adjusted model, this association remained significant (β = −0.7, SE = 0.2, OR = 3.7, 95% CI = 1.6–9.3, *P* = .0019). Other predictors, including age group, treatment facility for brain tumors, and surgical history of brain tumors, were not significantly associated with ACP experience (*P* > .05).

**Table 5.** Logistic Regression Analysis for Experience With ACP and the Frequency of Patients’ and Caregivers’ Discussions Regarding Treatment and Care

Model	Predictor	β^a^	SE	OR	95% CI	*P*-Value	*n*
Crude (Model 1)	Discussion with family^b^	−0.6	0.2	3.9	1.7–9.6	.001^c^	123
Adjusted (Model 2)	Discussion with family	−0.7	0.2	3.7	1.6–9.3	.0019^c^	122
	Age group	0.3	0.4	0.5	0.1–2.1	.386	
	Treatment facility for brain tumors	0.3	0.4	0.6	0.1–2.8	.4677	
	Surgical history of brain tumors	−0.5	0.6	2.6	0.4–52.1	.379	

Abbreviation: ACP, advance care planning.

^a^β, coefficient; CI, confidence interval; OR, odds ratio; SE, standard error.

^b^Discussion with family refers to the frequency of patients’ and caregivers’ discussions regarding treatment and care.

^c^Significant *P*-Values (*P* <.05) are marked with an asterisk (*).^c^

We further explored the patients’ and caregivers’ attitudes toward ACP through free-text responses, as summarized in Supplementary Tables 1 and 2. The patients expressed a wide range of attitudes toward ACP. Of the 109 patients, 61 provided free-text responses. Of these, 19 (17.4%) wanted to take a proactive approach, either engaging in shared decision-making with their families or wishing to decide for themselves. Many patients (*n* = 55, 50.5%) were willing to engage in ACP if their HCP suggested it, indicating the need for more triggers and information. Some patients (*n* = 19, 17.4%) understood the importance of ACP but were reluctant to pursue it due to emotional or situational factors, whereas others (*n* = 16, 14.7%) did not feel that it was necessary, often citing reasons such as financial burdens or a lack of understanding (Supplementary Table 1). The caregivers also showed varied attitudes toward ACP. Of the 91 caregivers, 52 provided free-text responses. Of these, 14 (15.4%) wanted to take a proactive approach. Many caregivers (*n* = 46, 50.5%) were willing to engage in ACP if the patient’s HCP suggested it, indicating the need for increased awareness and emotional readiness. Some caregivers (*n* = 20, 22.0%) understood the importance of ACP but were reluctant to pursue it because they feared it would cause anxiety or because they doubted that the patient’s preferences would be respected. Others (*n* = 11; 12.1%) did not feel that ACP was necessary, often because of previous decisions made within the family or because the patient was not in a condition to participate adequately in discussions (Supplementary Table 2).

## Discussion

This study provides an in-depth analysis of patients’ and caregivers’ knowledge, experiences, and preferences concerning ACP when managing PMBTs in Japan. The results provide significant insights into the different attitudes and preferences these groups have toward ACP. To ensure that both patients and caregivers are adequately informed and involved in ACP decision-making, careful consideration is required when introducing and discussing ACP in clinical practice.

Despite a relatively high willingness to engage in ACP (68.9% of the patients and 65.9% of the caregivers), actual knowledge about and experience with ACP were significantly lower. Only 12.3% of the patients and 10.7% of the caregivers knew about ACP, whereas 43.1% of the patients and 29.7% of the caregivers had experience with ACP. According to the Japanese MHLW report for 2023,^18^ 176 (5.9%) members of the public were familiar with ACP, and 898 (29.9%) had discussed it with family members or HCPs. This indicates that a higher percentage of patients with PMBTs and their caregivers have knowledge and experience concerning ACP than the general Japanese public. However, the gap between knowledge and willingness to participate suggests a critical need for more education and information from HCPs and the government.

In addition, the patients were significantly more likely than their caregivers perceived regarding wanting to participate in decisions about their treatment and care (96.8% vs. 82.5%, *P* < .001). This difference underscores the importance of directly involving patients in discussions about their care preferences and ensuring that they have the opportunity to actively participate in these decisions. Despite previous studies emphasizing the value of patient involvement in medical decision-making, which is associated with increased satisfaction and better alignment with patient values and preferences,^19^ significant gaps remain, especially in Japan. In conversations with HCPs, only 39% of patients with PMBTs received the same explanations regarding their exact medical condition and prognosis as their families.^20^ This may be due not only to the complex nature of PMBTs but also to a tendency among families to limit the details they divulge to patients in an effort to prevent emotional distress. Moreover, given the inevitable decline in the autonomy of patients with PMBTs, family members play a significant role in decision-making. HCPs often prioritize family preferences,^13^ and family preferences significantly influence patient participation in end-of-life discussions.^14^ Sometimes families do not involve patients in decision-making even when the patients are able to participate. These findings underscore the importance of patient-centered and family-involved shared decision-making, particularly in East Asian contexts such as that of Japan, where relational autonomy is respected and patients are often concerned about family opinions and potential burdens in decision-making.^1,21^

There was also a marked difference in preference about the timing of ACP. The caregivers were more likely to prefer initiating ACP at diagnosis (29.0% vs. 11.1%), whereas the patients were more likely to prefer initiating ACP at brain tumor recurrence (47.0% vs. 18.3%). This discrepancy indicates a potential gap in perception regarding when it is appropriate or beneficial to discuss ACP. The patients’ preference to wait until recurrence may reflect a desire to avoid prematurely confronting the implications of the diagnosis or it may reflect a lack of information about the severity of their condition. Only 39% of the patients were thoroughly informed about their condition or prognosis at the start of treatment,^20^ and thus, these patients may not recognize the need to discuss it earlier. Although disease explanations for patients at the start of treatment are not always sufficient, they gradually come to understand the importance of ACP through treatment and changes in their physical condition. Contrarily, caregivers may seek to ensure early planning to cope with uncertainty and stress. Although the timing of ACP discussions should be individualized, taking into account the readiness and preferences of both patients and their families,^22^ HCPs should first disclose information to, and elicit preferences from, patients themselves. Only then can they assess the appropriate time to initiate ACP. Future studies should explore how clinical factors, such as the time since diagnosis and tumor type, may influence the optimal timing for ACP discussions.

The logistic regression analysis further emphasized the role of family discussions in facilitating ACP. Consistent with our previous findings,^14^ which showed that active patient–family discussions were significantly associated with actual ACP experience, this analysis suggests that family involvement is a crucial component of the ACP process. HCPs can support this process by promoting open communication among family members and facilitating discussions.

The free-text responses highlighted additional nuances in attitudes toward ACP. More than half of the surveyed patients and caregivers indicated a willingness to engage in ACP if their HCPs suggested it, indicating a clear need for proactive engagement from HCPs. HCPs can assess patients’ and caregivers’ readiness for ACP discussions and provide appropriate triggers for initiating these conversations. The free-text responses also revealed barriers to ACP, including emotional reluctance, lack of understanding, and concerns about burdening family members. These barriers can be addressed through educational initiatives and supportive resources, which can help promote the adoption of ACP.^23^ Neurosurgeons and neuro-oncologists often play a pivotal role in guiding ACP discussions, yet many lack formal training in palliative care and communication skills.^24^ Communication tools such as the Serious Illness Care Program,^25^ VitalTalk,^26^ and Question Prompt List^27^ may help HCPs stay close to patients and caregivers, provide the necessary information, and offer ACP support.

Based on the present results, the committee on SCBTP under the JSNO—of which the present authors are part—created 3 types of ACP documents tailored to different stages of the clinical course: (1) before surgery, (2) at explanation of histological diagnosis after surgery, and (3) at brain tumor recurrence. We distributed these documents to patients and caregivers through the JSNO to disseminate more accurate information about ACP. These documents offer clear information, emotional support, and reassurance about the benefits and processes of ACP, helping address the barriers identified in this study. The documents are available only in Japanese. For more information, please visit the JSNO’s official website (https://www.jsn-o.com/).

Although this study provided important insights into patients’ and caregivers’ perspectives, experiences, and knowledge concerning ACP when managing PMBTs, it had some limitations. The sample was primarily from a tertiary care center in Japan, which may not reflect the cultural diversity and treatment varieties evident in the broader population of people with PMBTs. This limits the generalizability of the findings. However, the findings may be particularly relevant to East Asian contexts such as that of Japan, where relational autonomy is respected and patients are often concerned about family opinions and potential burdens in decision-making. In addition, the survey may have introduced a selection bias, both because the online format may have excluded older adults and people with brain dysfunction and because the survey was distributed by primary physicians, who may have excluded relevant participants if they deemed them incapable of responding. Furthermore, some caregivers may have been caring for individuals with cognitive dysfunction, which could have influenced their responses, particularly regarding ACP preferences. Some caregivers even noted in the free-text responses that their care recipients could no longer communicate effectively. Despite these limitations, the strengths of this study include its ability to gather voices from a relatively large number of patients and caregivers, individuals who offer a crucial perspective on ACP but who are frequently underrepresented in ACP research. Indeed, little direct data from patients with PMBTs exist on this matter. This study effectively quantified the differences between patients and caregivers in terms of their ACP preferences, and it provided a comprehensive view of the decision-making process by including caregivers’ perspectives.

## Conclusion

This study highlights the significant differences in patients’ and caregivers’ preferences, experiences, and attitudes concerning ACP when managing PMBTs in Japan. The patients favored greater involvement in decision-making and tended to delay ACP discussions until disease progression, whereas the caregivers preferred earlier planning. ACP knowledge was low among both patients and caregivers, while willingness to participate in ACP was high. Frequent discussions between patients and their family members play a crucial role in facilitating ACP, and both patients and caregivers are more likely to engage in ACP if encouraged to by HCPs. Increasing ACP awareness among patients with PMBTs, their caregivers, and HCPs, and encouraging HCPs to promote the early implementation of ACP, may improve the alignment between treatments and patient values and preferences, thereby enhancing patient-centered care. Future research should explore the underlying reasons for role and demographic differences in ACP preferences and develop targeted strategies to address these discrepancies.

## Supplementary material

Supplementary material is available online at *Neuro-Oncology Practice* (https://academic.oup.com/nop/).

npaf008_suppl_Supplementary_Appendix

npaf008_suppl_Supplementary_Tables

## Data Availability

The data that support the findings of this study are available to members of the Japan Society for Neuro-Oncology (JSNO) through the society’s official website: http://scbtp1.umin.jp/questionnaire.html.
