# Supplementary material for: Advance care planning in primary malignant brain tumors: Knowledge, experiences, and preferences of patients and caregivers
Source: Neurooncol Pract. 2025 Jan 21;12(4):732–42. doi: 10.1093/nop/npaf008 (PMC12349760; doi:10.1093/nop/npaf008)
Supplement: npaf008_suppl_Supplementary_Appendix [file npaf008_suppl_supplementary_appendix.docx]

**Appendix: Sample Questions from the Survey**

**Experience with ACP**
• Have you discussed treatments and care at the end of life with healthcare professionals and family members?

1. In-depth discussion
2. Some discussion
3. No discussion
   *Note: Responses 1 and 2 were grouped as "Yes," and response 3 was classified as "No" for analysis purposes.*

**Knowledge of ACP**
• Have you heard of the term "advance care planning (ACP)?"

1. I know the term and understand its meaning
2. I have heard of the term but do not fully understand its meaning
3. I have never heard of the term
   *Note: Response 1 was classified as "Knowledgeable," and responses 2 and 3 were classified as "Not knowledgeable" for analysis purposes.*

**Willingness to Engage in ACP**
• How do you feel about engaging in ACP with your family (or as a caregiver, with the patient) and healthcare professionals?

1. I want to actively take part
2. I would engage if suggested by healthcare professionals
3. I understand its importance, but I am reluctant
4. I do not feel it is necessary
   *Note: Responses 1 and 2 were grouped as "Willing," and responses 3 and 4 were grouped as "Not willing" for analysis purposes.*

**Attitude Toward Family Discussions on Treatment and Care**
• How frequently do you discuss treatment and care plans with your family (or as a caregiver, with the patient)?

1. Frequently
2. Sometimes
3. Rarely (only when prompted by healthcare professionals)
4. Never
   *Note: Responses 1 and 2 were grouped as "Proactive," and responses 3 and 4 were grouped as "Passive" for analysis purposes.*
