# Supplementary material for: Advance care planning in primary malignant brain tumors: Knowledge, experiences, and preferences of patients and caregivers
Source: Neurooncol Pract. 2025 Jan 21;12(4):732–42. doi: 10.1093/nop/npaf008 (PMC12349760; doi:10.1093/nop/npaf008)
Supplement: npaf008_suppl_Supplementary_Tables [file npaf008_suppl_supplementary_tables.docx]

**Supplementary Table 1. Patients’ Attitudes toward ACP**

| Category (total respondents *n* = 109) | Subcategory | Representative free-text responses (*n* = 61) |
| --- | --- | --- |
| Want to take a proactive approach (*n* = 19) | Want to engage in shared decision-making with family (*n* = 6) | I want to end my life happily with those who have been involved with me. |
|  |  | I want to ensure good communication with my family. |
|  | Want to decide for myself (*n* = 5) | I want to decide my own life. |
|  |  | I don’t want to leave it to others. |
|  | Want to prepare for the end of life (*n* = 4) | Because the end of life is inevitable, |
|  |  | I want to prepare for it. |
| Willing to engage if suggested by HCPs *^a^* (*n* = 55) | Want a trigger to initiate ACP (*n* = 6) | I would do it if suggested because there is no trigger. |
|  |  | Honestly, I want to engage in end-of-life planning. |
|  | Want information about prognosis and coping with the disease from HCPs (*n* = 6) | Because I can’t have a concrete discussion without detailed medical information from HCPs. |
|  |  | I want to know from HCPs how I can manage my brain tumor in the future and the symptoms that may occur. |
|  | Don't fully understand what ACP is (*n* = 6) | Because I have no idea. |
|  | Need support from HCPs (*n* = 5) | Because, with just family, we don't know how to talk about it. |
|  |  | I want to get advice from HCPs at some point. |
|  | Realized ACP is necessary for me (*n* = 4) | Because I had an opportunity to feel that “it’s too late” was not a far-off prospect. |
|  |  | Because I think end-of-life care is necessary for my disease. |
| Understand the importance but reluctant (*n* = 19) | Don't want to think about the end of life (*n* = 5) | I aim for a cure, and I don’t want to engage in negative aspects as it seems likely to lead to bad outcomes. |
|  |  | I avoid thinking about the future. |
|  | Concern about the emotional burden on family (*n* = 3) | I’m currently healthy, so my spouse, who is an important member of the meeting, wants to forget about difficult things for now. That’s the reason. |
|  |  | I’m worried it might shock the children. |
|  | Now is not the time to start ACP (*n* = 2) | Because nothing will happen immediately. |
|  | Want to leave it to the natural course (*n* = 1) | I want to leave it to the natural course of things because I accept what happens naturally. |
|  | Concern about physical burden on family (*n* = 1) | It’s important, but I feel bad about making my family or partner take time off work for the meeting. |
|  | Don't fully understand what ACP is (*n* = 1) | Because I don’t understand it well. |
|  | It’s a hassle (*n* = 1) | I find it bothersome. |
| Don’t feel it’s necessary (*n* = 16) | It’s not necessary (*n* = 2) | I don’t feel it’s necessary. |
|  | Concern about financial burden or solicitation (*n* = 2) | It seems like it would cost money. |
|  | Don’t know what to discuss in ACP (*n* = 1) | Because I don’t know what to talk about. |
|  | No sense of reality about death (*n* = 1) | There is no sense of reality about dying soon. |
|  | Don’t need support from HCPs (*n* = 1) | Because we talk enough as a couple. |
| *^a^* ACP, advance care planning; HCPs, healthcare professionals. *^b^* This table classifies patients’ attitudes toward ACP based on their responses to the question, “What do you think about ACP?” The total number of respondents was 109, of which 61 provided free-text responses. The categories represent the single-choice options in the questionnaire, while the subcategories were derived from a qualitative analysis of the content of the open-ended responses. | | |

**Supplementary Table 2. Caregivers’ Attitudes toward ACP**

| Category (total respondents *n* = 91) | Subcategory | Representative free-text responses (*n* = 52 ) |
| --- | --- | --- |
| Want to take a proactive approach (*n* = 14) | Want to plan a future with the patient and family to ensure the patient lives a fulfilled life (*n* = 5) | I want the patient to live a satisfactory life. |
|  |  | I want to set a clear outlook with my family to ensure we can all face the end in the best possible way. |
|  | Recognize the need for shared decision-making (*n* = 4) | I want healthcare professionals to understand the patient’s wishes. |
|  |  | Although there were differing opinions, we understood each other’s feelings. I feel we can move forward with a new perspective on our future lives. I want to pass this on to my children. |
|  | Want to think about life and death by themselves (*n* = 3) | Because I want to decide my own way of living and dying. |
| Willing to engage if suggested by HCPs *^a^* (*n* = 46) | Don’t fully understand what ACP is (*n* = 7) | Because I don’t know about it, I would like to be taught if necessary. |
|  |  | Because we lack professional knowledge about healthcare, we don't know when and what to propose from our side. |
|  | Not emotionally ready to promote ACP by themselves (*n* = 4) | It’s difficult to proceed proactively as it seems pessimistic about the future. |
|  |  | I can’t think about it yet. |
|  | Need mediation by HCPs in discussions (*n* = 4) | When it comes to discussing end-of-life matters, it’s hard to be proactive if it’s just the person involved. |
|  |  | It might be easier to talk about difficult things with someone else present. |
|  | Want to know the patient's preferences (*n* = 3) | I want to hear the patient’s preferences. |
|  |  | Given the limited time, it’s important to confirm the patient’s preferences. |
|  | Want to participate according to the patient’s preferences (*n* = 3) | If the patient thinks it’s necessary, I want us all to discuss it. |
| Understand the importance but reluctant (*n* = 20) | Don’t want to make the patient anxious (*n* = 4) | Because doing so would cause anxiety in the patient. |
|  |  | The patient is currently in post-treatment observation, so I don’t want to talk about life and death. |
|  | Can’t communicate well with the patient (*n* = 3) | Because the patient (father) is not fully conscious. |
|  |  | The patient doesn’t want it. Especially now, he/she can’t talk and it’s too late for long discussions. |
|  | Doubt whether the patient’s preferences will be respected (*n* = 2) | I don’t want it to be led by doctors or HCPs. I suspect that hospital circumstances and bed counts will be factored in. |
|  | Preferences change with circumstances (*n* = 2) | It’s fine to decide when the time comes. |
|  | Too tired from caregiving to think about it (*n* = 1) | I’m too tired from caregiving to think about it. |
|  | Don't need support from HCPs (*n* = 1) | Because everyone has their own way of dealing with life. |
|  | Don’t fully understand what ACP is (*n* = 1) | I don’t know about ACP. |
| Don't feel it’s necessary (*n* = 11) | Doubt whether the patient’s preferences will be respected (*n* = 2) | Because I doubt whether the patient’s preferences will truly be respected. |
|  | Already decided between the patient and their family (*n* = 1) | Because we have already decided to spend the final days at home with the family, there is no need for special discussions as we don’t have advanced life-sustaining equipment at home. |
|  | The patient is not in a condition to discuss (*n* = 1) | Because the patient is no longer able to communicate sufficiently. |
|  | Don’t fully understand what ACP is (*n* = 1) | Because I don’t understand the content. |
| *^a^* ACP, advance care planning; HCPs, healthcare professionals. *^b^* This table classifies caregivers’ attitudes toward ACP based on their responses to the question, “What do you think about ACP?” The total number of respondents was 91, of which 52 provided free-text responses. The categories are the choices in the questionnaire, and the subcategories are derived from the content of the open-ended responses.  *^C^* The total number of respondents was 91, of which 52 provided free-text responses. For detailed explanations regarding the categories and subcategories, please refer to the footnotes in Appendix Table 1 (patients). | | |
